# High Expression of *PARP1* in Tumor and Stroma Cells Predicts Different Prognosis and Platinum Resistance in Patients With Advanced Epithelial Ovarian Cancer

**DOI:** 10.3389/fonc.2022.931445

**Published:** 2022-07-07

**Authors:** Wei-Wei Zuo, Chun-Fang Zhao, Yan Li, Hai-Yan Sun, Guo-Ming Ma, Yue-Ping Liu, Shan Kang

**Affiliations:** ^1^ Department of Gynecology, Fourth Hospital, Hebei Medical University, Shijiazhuang, China; ^2^ Department of Gynecology, Tangshan People’s Hospital, Tangshan, China; ^3^ Department of Histoplasty and Embryology, Hebei Medical University, Shijiazhuang, China; ^4^ Department of Molecular Biology, Fourth Hospital, Hebei Medical University, Shijiazhuang, China; ^5^ Department of gastrointestinal surgery, Tangshan People’s Hospital, Tangshan, China; ^6^ Department of Pathology, Hebei Medical University, Fourth Hospital, Shijiazhuang, China

**Keywords:** advanced epithelial ovarian cancer, *PARP1*, cytokeratin, platinum resistance, prognosis

## Abstract

**Objective:**

This study aimed to explore the roles of PARP1 mRNA and protein expression in platinum resistance and prognosis of EOC patients, and reveal the different roles of PARP1 protein in epithelial tumor and stroma cells.

**Methods:**

The *PARP1* mRNA expression of the EOC tissues was examined by RT-qPCR. The impacts of *PARP1* expression on prognosis were measured by Kaplan-Meier and Cox regression. Receiver operating characteristic (ROC) curve analysis was employed for calculating the diagnostic value of *PARP1* on platinum resistance. The microarray of formalin-fixed, paraffin-embedded (FFPE) tissues was processed for multiplex immunofluorescence to detect the protein levels of PARP1 and cytokeratin (CK).

**Results:**

The *PARP1*mRNA expression of EOC patients was higher in the platinum-resistant group compared with the sensitive group (P<0.01). Kaplan-Meier analysis demonstrated that high *PARP1* mRNA expression was associated with poor survival of EOC patients. In Cox regression analyses, high *PARP1* mRNA expression independently predicted poor prognosis (P=0.001, HR=2.076, 95%CI=1.373-3.140). The area under the ROC curve of *PARP1* mRNA for predicting the platinum resistance in EOC patients was 0.649, with a sensitivity of 0.607 and specificity of 0.668. Furthermore, the protein expression of *PARP1* was higher in the platinum-resistant group than in the sensitive group (P<0.01) and associated with a worse prognosis. Additionally, according to CK labeling, we observed that enhanced expression of *PARP1* in the CK+ region was associated with platinum resistance and lower survival, but in CK- region, it predicted a good prognosis and platinum sensitivity.

**Conclusion:**

*PARP1* may be a potential biomarker to predict platinum resistance and prognosis for EOC patients, exerting different roles on epithelial tumor and stromal cells.

## Introduction

Epithelial ovarian cancer (EOC) is the most common type of ovarian cancer and the most lethal gynecological malignancy (1). Due to the lack of initial symptoms and sensitive screening methods, approximately 70% of EOC patients are diagnosed at an advanced stage (2). The standard treatment strategy for patients with advanced EOC is primary debulking surgery followed by platinum-based chemotherapy. Because of chemotherapy resistance (3) and late diagnosis, the 5-year survival rate in patients with advanced EOC is below 40% (4, 5). Currently, *PARP1* inhibitors have emerged as one of the successful novel approaches to targeted ovarian cancer treatment (6, 7).

*PARP1* is a multifunctional nuclear enzyme found in most eukaryotic cells, which is activated by recognizing the structurally damaged DNA fragments and is considered a receptor of DNA damage (8, 9). The enhancement of *PARP1* expression and activity can effectively repair the DNA damage caused by platinum drugs (10), leading to a decrease in the efficacy of chemotherapy on tumor cells (11). Inhibition of *PARP1* expression and activity has been shown to reverse the resistance to chemotherapy and radiotherapy in multiple tumors, such as breast cancer and pancreatic cancer (12, 13). In ovarian cancer, several studies suggested that high expression of *PARP1* may be a potential marker for predicting poor prognosis and platinum resistance of the patients (14, 15). At present, some studies have shown that *PARP1* inhibitors can significantly prolong the PFS and OS of patients with epithelial ovarian cancer and breast cancer (16, 17). Nonetheless, different responses to this treatment were observed among individuals (18, 19). The underlying reason is still unclear.

In the present study, the role of *PARP1* mRNA and protein expression in platinum resistance and clinical prognosis of EOC was investigated by conducting a hospital-based case-control study. Moreover, the different roles of *PARP1* expression in tumor cells and stroma cells were revealed *via* multiplex immunofluorescence assay, and *PARP1* expression in stroma cells may affect the response to *PARP1* inhibitors.

## Materials and Methods

### Tissue Samples Collection

A total of 143 advanced EOC tumor samples were collected in this study from the Fourth Hospital, Hebei Medical University, between October 2012 and November 2015. During the same period, normal ovarian tissues (n=20) were collected from patients who underwent hysterectomy for benign uterine disease. According to the International Federation of Gynecology and Obstetrics (FIGO) (2014), all EOC samples were stage III or IV. This study was approved by the Ethics Committee of the Fourth Hospital of Hebei Medical University (NO. 2017ME96) and signed informed consents were obtained from each patient.

Based on the platinum-free interval (PFI), patients were divided into the platinum-resistant group (Resistant group) and platinum-sensitive group (Sensitive group). Briefly, patients with PFI < 6 months were considered platinum-resistant, whereas patients with PFI >6 months were deemed platinum-sensitive (20). All participants were regularly followed up for 5 years. Progression-free survival (PFS) and overall survival (OS) were assessed to analyze the survival status of EOC patients.

The inclusion criteria were patients who 1) were diagnosed as primary EOC by histopathological examination; 2) women of any age; 3) had not received chemotherapy or radiotherapy prior to radical resection. The exclusion criteria were patients who 1) had other types of cancers; 2) had a past record of therapy, including chemotherapy or radiation before surgery.

### Quantitative Real-Time PCR

Total RNA from tissue samples was extracted by the TRIzol-chloroform extraction method (Invitrogen, Carlsbad, CA, USA). Subsequently, cDNA synthesis was conducted using Revert Aid First Strand cDNA Synthesis Kit (Thermo Fisher Scientific, Waltham, MA, USA). The RT-qPCR reaction was performed using SYBR-Green II Premix (Takara, Dalian, China) with the ABI 7500 detection system (Applied Biosystems, Carlsbad, CA, USA). The reaction conditions were 95°C for 30 s, and 40 cycles of 95°C for 5 s, 60°C for 30 s, and 72°C for 5s. The primer sequences were synthesized by Sangon Biotech Co., Ltd. (Shanghai, China) as follows: *PARP1* (forward)5′-AGTATGCCAAGTCCAACAGAAGTACG-3′,(reverse)5′-CCAGCGGTCAATCATGCCTAGC -3′; GAPDH (forward) 5′-ACCACAGTCCATGCCATC AC-3′, (reverse) 5′-CATGTGACA GAAGTACG-3′. GAPDH was termed internal control.

### Gene Expression Analysis of PARP1

GEPIA (http://gepia.cancer-pku.cn/index.html) is an analytical tool using a standard processing pipeline and consist of thousands of tumors and normal tissue samples data. In this study, student’s t-test was used to generate a p-value for the *PARP1* expression profiles through GEPIA combined with the Cancer Genome Atlas (TCGA) dataset and the Genotype-Tissue Expression (GTEx) projects. The cut-off value of log2FC was set as 1, and P value was set to 0.01.

### Establishment of Tissue Microarray

Formalin-fixed, paraffin-embedded (FFPE) tissues from 133 tumor samples (10 FFPE tissues missing) were processed for establishing the tissue microarray. Briefly, highly representative areas were designated by pathologists *via* reviewing the HE staining sections and then a tissue microarray was produced using a tissue arrayer (Beecher Instruments, Silver Spring, MD, USA). Subsequently, the tissue microarray was sectioned continuously (4 μm) and then heated in the oven at 63°C for 1h for immunohistochemistry and multiplex immunofluorescence assays.

### Immunohistochemistry Assay

An immunohistochemistry assay was utilized to detect the *PARP1* levels in the tissue microarray sections. Briefly, 4 μm slides were treated with 3% hydrogen peroxide-containing methanol, followed by incubation with 10% goat serum for blocking. After washing, the slides were incubated with anti-PARP1 antibody (1:2000, ab227244, Abcam, Cambridge, UK) and then EnVision™ FLEX/HRP (Dako, Glostrup, Denmark). Subsequently, EnVision™ FLEX DAB kit (Dako, Glostrup, Denmark) and hematoxylin were applied for staining. The PARP1 expression was analyzed based on the number of positive cells per mm2 by the imaging system (Vectra Polaris, PerkinElmer, Waltham, MA). The median of the positive cells per mm2 was considered as an watershed of positive or negative individuals.

### Multiplex Immunofluorescence Assay

Slides of tissue microarray were heated in the oven at 63°C for 1 h, dewaxed, boiled in a citrate buffer, and repaired for 15-20min with low heat. After cooling and washing, the slides were incubated with the following primary antibodies in order: anti- *PARP1* antibody (1:2000, ab227244, Abcam, Cambridge, UK), anti-pan-CK (PA125, Baidao Medical Technology, Suzhou, China). Subsequent secondary antibody (Dako, Glostrup, Denmark) was added and incubated at room temperature for visualization. Staining with Opal dye solution (1:100) was repeated 5 times. One cycle of antibody staining consisted of blocking, incubation with first primary antibody (*PARP1)* and secondary antibody, Opal dye, and removal of bound antibody. The abovementioned cycle was repeated for the second primary antibody (CK). The slides were counter-stained with DAPI, and then images were captured by an automatic quantitative pathology imaging system (Vectra Polaris, PerkinElmer, Waltham, MA). The *PARP1* -positive cells were counted in positive and negative regions based on CK expression (CK+ region and CK- region). The median of the CK positive cells per mm2 was considered as the standard of division on CK+ and CK-.

### Statistical Analysis

Data were analyzed using the SPSS 21.0 software package (SPSS Inc., Chicago, IL, USA). Kaplan-Meier method with the log-rank test was utilized to analyze the *PARP1* expression on survival rates. Enumeration data were analyzed by χ2 test, and the differences in measurement data were compared with Student’s t-test between the two groups. Receiver operating characteristic (ROC) curve analysis was employed for calculating the diagnostic value of *PARP1.* The Cox proportional hazards regression model was used to perform univariate and multivariate survival analyses. P<0.05 was considered as a significant difference.

## Results

### Baseline Characteristics

As shown in **Table 1**, 143 clinical samples were analyzed, with the median age of patients being 55 years old (ranged 34-75 years). In terms of pathological type, there were 82 cases (57.3%) of serous carcinoma, 26 (18.2%) of endometrioid carcinoma, 22 (15.4%) of mucinous carcinoma, and 13 (9.1%) of other types. According to FIGO staging, 134 cases (93.7%) were in stage III and 9 cases (6.3%) in stages IV. Of the 143 patients with a known grade, 22 (15.4%) were G1 grade, 32 (22.4%) were G2 grade, and 89 (62.2%) were G3 grade.

**Table 1:** Baseline characteristics of patients with advanced epithelial ovarian cancer.

Characteristics	No. of patients (n = 143)
Histology (n, %)	
Serous carcinoma	82 (57.3%)
Endometrioid carcinoma	26 (18.2%)
Mucinous carcinoma	22 (15.4%)
Mixed type	13 (9.1%)
FIGO stage (n, %)	
III	134 (93.7%)
IV	9 (6.3%)
Histological grade (n, %)	
G1	22 (15.4%)
G2	32 (22.4%)
G3	89 (62.2%)
Tumor residual size (n, %)	
0 cm	60 (41.9%)
≤ 1cm	64 (44.8%)
>1cm	19 (13.3%)
Tumor size (n, %)	
≤ 10cm	62 (43.4%)
>10cm	81 (56.6%)
Number of chemotherapy (n, %)	
≤4	42 (29.4%)
>4	101 (70.6)%

FIGO, International Federation of Gynecology and Obstetrics.

### *PARP1* Expression Was Up-Regulated and Relevant to Platinum Resistance

**Figure 1:**
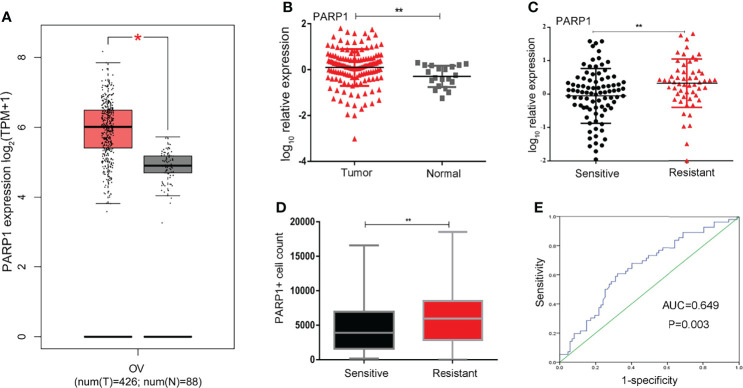
PARP1 expression in ovarian tumor and normal tissue and in specimens from sensitive and resistant patients. **(A)**The differential expression of PARP1 in ovarian tumor and normal ovarian tissues was analyzed by GEPIA. Red colour means ovarian cancer tissues and grey colour means normal tissues; **(B)** The mRNA expression of PARP1 in EOC samples (Tumor) and normal ovarian tissues (Normal) was tested by qRT-PCR; **(C)** qRT-PCR was utilized to examine the mRNA expression of PARP1 between Resistant and Sensitive groups; **(D)** The protein level of PARP1 was detected by immunofluorescence assay of microarray in paraffin-embedded tissue; **(E)** ROC analysis was used to investigate diagnostic value of PARP1 on platinum resistance of EOC. *P < 0.05, **P < 0.01; EOC, Epithelial ovarian cancer.

We identified the expression levels of *PARP1* in OC (426 cases) and normal ovarian (88 cases) tissues by GEPIA website which based on TCGA and GTEx database. As illustrated in **Figure 1A**, *PARP1* expression in ovarian tumor tissues was approximately 1.4 times of the normal ovarian tissues. Consistently, we found that *PARP1* expression in EOC samples was up-regulated (**Figure 1B**). The relationship between *PARP1* expression and platinum resistance was further explored, and results revealed that the Resistant group had a significantly higher *PARP1* expression than the Sensitive group (P<0.01) (**Figure 1C**). Similarly, the immunofluorescence assay of the tissue microarray revealed a strong correlation between *PARP1* expression and platinum resistance (**Figure 1D**, **Table 2**). Spearman’s correlation analysis revealed that there was a significant positive connection between *PARP1* mRNA expression and protein level (P = 0.00, r = 0.41). To investigate the diagnostic value of *PARP1* in platinum resistance in EOC, ROC analysis demonstrated that the area under the curve (AUC) was 0.649 (95% CI, 0.558-0.741) and the cut-off value was 1.78 (sensitivity=0.607, specificity=0.678, Youden’s index=0.285) (**Figure 1E**). The aforementioned findings suggest that *PARP1* expression was up-regulated and was associated with platinum resistance.

**Table 2:** Associations of platinum-based chemotherapy resistance with PARP1 protein expression.

PARP1^+^cell count	High expression (n, %)	Low expression (n, %)	χ^2^	P value
Sensitive group	31 (41.33)	44 (58.67)	4.729	0.023
Resistant group	35 (60.34)	23 (39.66)		

### High Expression of *PARP1* Was Associated With Poor Prognosis in EOC Patients

**Figure 2:**
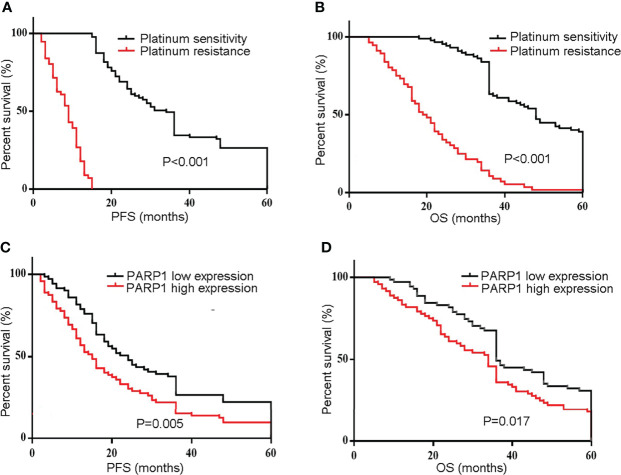
The mRNA expression of PARP1 was up-regulated in EOC patients and associated with poor prognosis. **(A, B)** The PFS and OS in resistant group and sensitive group were compared using Kaplan-Meier analysis; **(C, D)** The mRNA expression of PARP1 influencing the PFS and OS. PFS, Progression-free survival; OS, Overall survival; EOC, Epithelial ovarian cancer.

Platinum resistance is one major reason for mortality in EOC patients. First, Kaplan-Meier analysis was used to compare the PFS and OS of the Resistant group and the Sensitive group. Furthermore, the PFS and OS in the Resistant group were significantly poorer than the Sensitive group (P<0.001) (**Figures 2A,B**). Additionally, the median PFS was 24 months in the low *PARP1* group and 15 months in the high *PARP1* group (P=0.005), while the median OS was 36 months in the low *PARP1* group and 34 months in the high *PARP1* group. There were notable differences in PFS and OS between the two groups (P=0.005, P=0.017) (**Figures 2C,D**).

**Figure 3:**
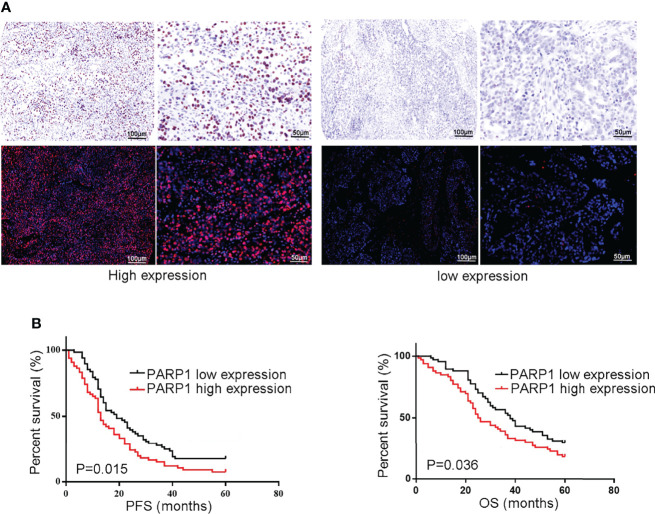
The protein level of PARP1 was up-regulated in microarray of formalin-fixed, paraffin-embedded tissues from tumor samples and correlated to poor prognosis. **(A)** Representive images of PARP1 with high expression (brown and red; Scar bar=100 and 50μm); **(B)** The protein level of PARP1 influencing the PFS and OS was measured by Kaplan-Meier method. PFS, Progression-free survival; OS, Overall survival; EOC, Epithelial ovarian cancer.

Similarly, a microarray of FFPE tissues from 133 tumor samples revealed that patients with high *PARP1* levels had lower PFS and OS (**Figure 3**). Furthermore, the Cox regression model was utilized to evaluate the prognosis factors. *PARP1* expression was one of the independent risk factors affecting the prognosis of EOC patients (P=0.001, HR=2.076, 95%CI =1.373-3.140), as displayed in **Table 3**. Collectively, *PARP1* may be closely associated with poor prognosis in EOC patients.

**Table 3:** Association of variables with the 5-year PFS and OS of epithelial ovarian cancer patients by Univariate and Multivariate Cox proportional hazards models.

	OS			PFS		
	HR	(95% CI)	P value	HR	(95% CI)	P value
Age						
(≤55 vs >55)	0.99	0.637-1.539	0.965	0.816	0.540-1.230	0.33
Stage						
(IV vs III)	1.532	0.592-3.966	0.379	1.96	0.892-4.306	0.094
Grade						
(G2 vs G1)	3.748	0.999-14.067	0.05	1.925	0.677-5.476	0.219
Grade						
(G3 vs G1)	2.934	0.842-10.219	0.091	1.95	0.751-5.064	0.17
Tumor size						
(≤10cm vs >10cm)	0.782	0.481-1.269	0.575	2.934	0.842-10.219	0.091
Tumor residual size						
(≤1cm vs 0cm)	2.019	1.205-3.382	0.008	1.882	1.189-2.979	0.007
Tumor residual size						
(>1cm vs 0cm)	4.018	2.074-7.784	0.000	3.59	1.959-6.580	0.000
PARP1 expression						
(High vs low)	2.398	1.507-3.814	0.000	2.076	1.373-3.140	0.001

PFS, Progression-free survival; OS, Overall survival; HR, Hazard ratio; CI, Confidence interval.

### *PARP1* Predicted Prognosis and Platinum Resistance in Epithelial Tumor Cells and Nonepithelial Cells

**Figure 4:**
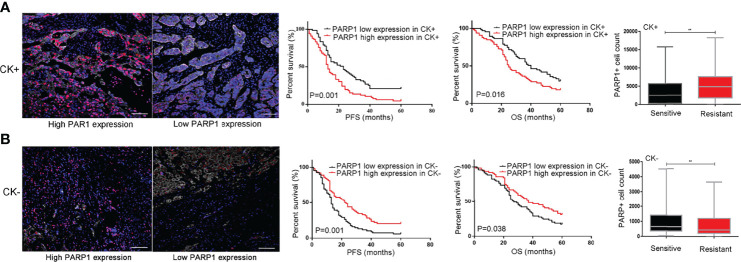
PARP1 predicted prognosis and platinum resistance in epithelial tumor cells and stromal cells. **(A)** High protein level of PARP1 (red) in CK+ region (white) indicated EOC patients with poor prognosis and platinum resistance (Scar bar=50μm); **(B)** High protein level of PARP1 in CK- region indicated EOC patients with good prognosis and platinum sensitivity (Scar bar=50μm). CK, Cytokeratins; PFS, Progression-free survival; OS, Overall survival; EOC, Epithelial ovarian cancer. **P < 0.01

The tumor microenvironment and tumor-stromal interactions exert pivotal roles on tumor development. In this study, CK labeling is utilized to distinguish epithelial tumor cells from stroma cells in EOC tissues. The impact of *PARP1* expression in epithelial tumor cells and stroma cells on platinum resistance and prognosis was investigated using microarray analysis. As presented in **Figure 4A**, increased *PARP1* expression in the CK+ region was associated with a decreased survival rate (P<0.05). Additionally, the *PARP1* levels of the CK+ region in the Resistant group were significantly higher than the Sensitive group (P<0.01); the *PARP1* level in the CK+ region was positively correlated with platinum resistance (**Table 4**). These findings suggested that high *PARP1* expression in epithelial tumor cells is associated with poor prognosis and platinum resistance in EOC patients.

**Table 4:** Associations of platinum-based chemotherapy resistance with PARP1 protein level of CK+ and CK- region.

PARP1^+^ cell count		High expression (n, %)	Low expression(n, %)	χ^2^	P value
CK+	Sensitive group	31 (41.33)	44 (58.67)	4.729	0.023
	Resistant group	35 (60.34)	23 (39.66)		
CK-	Sensitive group	42 (56.00)	33 (44.00)	4.277	0.029
	Resistant group	22 (37.93)	36 (62.07)		

CK, Cytokeratins.

In CK- region, Kaplan-Meier analysis showed that patients with high *PARP1* expression had longer PFS and OS (P=0.001, P=0.038) (**Figure 4B**). Besides, we observed that the *PARP1* level of the CK- region was significantly higher in the Sensitive group than the Resistant group (P<0.01). Correlation analysis revealed a negative association between *PARP1* expression in CK- region and platinum resistance (**Table 4**). The aforementioned findings demonstrate that high *PARP1* expression in stroma cells predicted a good prognosis and platinum sensitivity for EOC patients.

Taken together, *PARP1* exerted different effects on epithelial tumors and the surrounding stroma cells in modulating EOC progression.

## Discussion

In this study, qPCR and multiplex immunofluorescence assay were applied to analyze the relationship between *PARP1* expression and platinum resistance and clinical prognosis in patients with EOC. Our findings indicate that high expression of *PARP1* may result in platinum resistance in patients with EOC, resulting in significantly shortened PFS and OS.

Numerous studies have indicated that the chemotherapeutic mechanism of platinum compounds is known to produce DNA crosslinks (inter- and intra-strand) and induce DNA double-strand breaks and cell apoptosis (21). However, tumor cells respond to these threats by activating multiple pathways of DNA repair (22). As a DNA damage sensor and signal transducer (23), *PARP1* is activated by DNA breaks and participates in their repair, playing a critical role in the survival of cancer cells and influencing platinum sensitivity (24). Consistent with these findings, several studies have reported that *PARP1* has a crucial impact on chemotherapeutic resistance in various cancers (25, 26). In ovarian cancer, some clinically relevant trials have reported that high expression of *PARP1* is associated with drug resistance and poor prognosis. For example, Zhang et al. (27) have revealed that high expression of *PARP1* protein is associated with drug resistance and shorter overall survival. Furthermore, a study published by Barnett JC revealed that high expression of *PARP1* in serous ovarian cancers is associated with worse OS (28). In addition, another study showed that high expression of *PARP1* evaluated by immunohistochemically in 174 sporadic high-grade serous carcinoma patients is associated with a poor outcome when combined with either high or low *BRCA* expression (29). Similarly, clinical data from 86 cases of high-grade EOC were analyzed, and it was discovered that the absence of *PARP1* expression as determined by immunohistochemistry predicted superior PFS and chemotherapy sensitivity in patients with ovarian cancer treated with adjuvant platinum-based chemotherapy (30). These results provide strong evidence that high expression of *PARP1* may have a critical role in chemotherapy resistance and a worse prognosis in EOC. Our study also concluded that increased *PARP1* mRNA and protein expression levels were associated with platinum resistance and a poor clinical outcome in patients with EOC. The possible molecular mechanism of the influence of *PARP1* on platinum resistance and prognosis has also been studied. In addition to clinical studies, a number of *in vitro* studies have shown that knockdown of *PARP1* can inhibit the proliferation of tumor cells and reverse the platinum resistance. For instance, when adjusted by miRNA let-7e, high *PARP1* expression was reported to modulate the cisplatin sensitivity in EOC cells (31). Another *in vitro* study showed that the mRNA level of *PARP1* was significantly regulated by miR-216b, and the overexpression of *PARP1* mRNA can significantly restrain cisplatin sensitivity in EOC cells (32). Taken together, increased *PARP1* expression, whether at the mRNA or protein level, *in vitro* or *in vivo*, correlates with a poor platinum response and survival. Hence, *PARP1* is a potential biological target in the treatment of EOC, especially in the presence of platinum resistance.

Remarkably, multiple studies have confirmed that *PARP1* inhibitors can improve poor prognosis in ovarian cancer. In recent years, it has been approved for the maintenance therapy of patients with EOC after the first-line therapy and the treatment of platinum-sensitive recurrent ovarian cancer patients. However, there were still a significant number of patients with an unexplained lack of improvement in the clinical application of *PARP1* inhibitors. According to current research, the tumor microenvironment may play an important role in tumor genesis and development (33, 34). Some past research has shown that the tumor microenvironment is made up of more than just tumor cells and also involves stromal cells and infiltrating cells of the immune system, which are likely affected by *PARP1* inhibition (35). In this study, we analyzed the expression levels of *PARP1* protein in tumor cells (CK+) and stroma cells (CK-) from EOC tissues using multiplex immunofluorescence assay. Surprisingly, our results from multiplex immunofluorescence assay showed that high expression of *PARP1* in stroma cells significantly prolonged PFS and OS in patients with EOC, which was inversed in tumor cells. Interestingly, *PARP1* overexpression led to opposite effects in tumor cells and stroma cells. The enhanced expression of *PARP1* in stroma cells may change the tumor microenvironment, which may explain the variation in the efficacy of *PARP1* inhibitors in clinical application. In the present study, *PARP1* expression in stroma cells of EOC may activate non-tumor cell repair to alter the tumor microenvironment, thereby enhancing the immune killing effect on tumor cells. A previous study has demonstrated that among various normal tissues, *PARP1* expression was highest in lymphatic tissues, indicating that it may participate in body immunity (36). Consistent with the important role of *PARP1* in stroma cells, Natasha Kyprianou et al. (37) have indicated the loss of *PARP1* expression in the (stromal) mesenchymal cells in prostate tumors after radiotherapy is associated with biochemical recurrence and poor response to radiotherapy. Therefore, we speculated that the disparity of *PARP1* inhibitor effectiveness in different EOC patients could be attributed to the difference in *PARP1* expression in stroma sections.

However, this study also has limitations. The samples were collected from a single center. In the EOC tumor-associated stromal microenvironment, *PARP1* expression was not observed in different specific cell types. In the future, it is necessary to confirm this conclusion through a large sample, multi-center clinical study focusing on the effect of *PARP1* expression in the CK- region on platinum resistance and prognosis.

Overall, those findings revealed that *PARP1* might be a potential biomarker to predict platinum resistance and prognosis in patients with advanced EOC; however, its specific mechanism needs to be further studied.

## Data Availability Statement

The original contributions presented in the study are included in the article material. Further inquiries can be directed to the corresponding author.

## Ethics Statement

The studies involving human participants were reviewed and approved by the Ethics Committee of the Fourth Hospital of Hebei Medical University (NO. 2017ME96). The patients/participants provided their written informed consent to participate in this study.

## Author Contributions

Administrative support: W-WZ, SK, C-FZ and YL. Provision of study materials or patients: W-WZ and H-YS. Collection and assembly of data: W-WZ and H-YS. Data analysis and interpretation: W-WZ, G-MM, Y-PL, and YL. All authors contributed to the article and approved the submitted version.

## Funding

This work was supported by grants from the Natural Science Foundation of Hebei Province (Grant number: H2020206385)

## Conflict of Interest

The authors declare that the research was conducted in the absence of any commercial or financial relationships that could be construed as a potential conflict of interest.

## Publisher’s Note

All claims expressed in this article are solely those of the authors and do not necessarily represent those of their affiliated organizations, or those of the publisher, the editors and the reviewers. Any product that may be evaluated in this article, or claim that may be made by its manufacturer, is not guaranteed or endorsed by the publisher.
