# Corrigendum: High expression of PARP1 in tumor and stroma cells predicts prognosis and platinum resistance in patients with advanced epithelial ovarian cancer

**DOI:** 10.3389/fonc.2023.1218746

**Published:** 2023-06-27

**Authors:** Wei-Wei Zuo, Chun-Fang Zhao, Yan Li, Hai-Yan Sun, Guo-Ming Ma, Yue-Ping Liu, Shan Kang

**Affiliations:** ^1^ Department of Gynecology, Fourth Hospital, Hebei Medical University, Shijiazhuang, China; ^2^ Department of Gynecology, Tangshan People’s Hospital, Tangshan, China; ^3^ Department of Histoplasty and Embryology, Hebei Medical University, Shijiazhuang, China; ^4^ Department of Molecular Biology, Fourth Hospital, Hebei Medical University, Shijiazhuang, China; ^5^ Department of gastrointestinal surgery, Tangshan People’s Hospital, Tangshan, China; ^6^ Department of Pathology, Hebei Medical University, Fourth Hospital, Shijiazhuang, China

**Keywords:** advanced epithelial ovarian cancer, *PARP1*, cytokeratin, platinum resistance, prognosis

In the published article, there was an error in the Funding statement. The Grant number for the Natural Science Foundation of Hebei Province was displayed as “H20202063”. The correct Funding statement appears below.

The correct Funding statement is “This work was supported by grants from the Natural Science Foundation of Hebei Province (Grant number: H2020206385).”

The authors apologize for this error and state that this does not change the scientific conclusions of the article in any way. The original article has been updated.

